# Efficacy and Safety of CT-P13 as First- and Second-Line Treatment in Patients with Ankylosing Spondylitis

**DOI:** 10.3390/jcm13237266

**Published:** 2024-11-29

**Authors:** Sadettin Uslu, Semih Gülle, Gercek Sen, Sedat Capar, Soner Senel, Ediz Dalkılıc, Servet Akar, Süleyman Serdar Koca, Abdurrahman Tufan, Ayten Yazici, Sema Yilmaz, Nevsun Inanc, Merih Birlik, Dilek Solmaz, Ayse Cefle, Berna Goker, Haner Direskeneli, Servet Yolbas, Niels Steen Krogh, Neslihan Yilmaz, Sükran Erten, Cemal Bes, Ozgül Soysal Gündüz, Mehmet Akif Oztürk, Seminur Haznedaroglu, Sule Yavuz, Fatos Onen, Ismail Sari

**Affiliations:** 1 Division of Rheumatology, School of Medicine, Celal Bayar University, 45140 Manisa, Turkey; ozgul.gunduz@cbu.edu.tr; 2 Division of Rheumatology, School of Medicine, Dokuz Eylul University, 35220 Izmir, Turkey; gulle.semih@deu.edu.tr (S.G.); gercek.can@deu.edu.tr (G.S.); birlikm@deu.edu.tr (M.B.); fatos.onen@deu.edu.tr (F.O.); ismail.sari@deu.edu.tr (I.S.); 3 Department of Statistics, Faculty of Science, Dokuz Eylul University, 35390 Izmir, Turkey; sedat.capar@deu.edu.tr; 4 Division of Rheumatology, School of Medicine, Erciyes University, 38030 Kayseri, Turkey; ssenel@erciyes.edu.tr; 5 Division of Rheumatology, School of Medicine, Uludag University, 16285 Bursa, Turkey; edalkilic@uludag.edu.tr; 6 Division of Rheumatology, School of Medicine, Kâtip Celebi University, 35620 Izmir, Turkey; servet.akar@ikcu.edu.tr (S.A.); dilek.solmaz@ikcu.edu.tr (D.S.); 7 Division of Rheumatology, School of Medicine, Firat University, 23119 Elazig, Turkey; sskoca@firat.edu.tr; 8 Division of Rheumatology, School of Medicine, Gazi University, 06570 Ankara, Turkey; atufan@gazi.edu.tr (A.T.); bgoker@gazi.edu.tr (B.G.); makifozturk@gazi.edu.tr (M.A.O.); seminur@gazi.edu.tr (S.H.); 9 Division of Rheumatology, School of Medicine, Kocaeli University, 41001 Kocaeli, Turkey; ayten.yazici@kocaeli.edu.tr (A.Y.); ayse.cefle@kocaeli.edu.tr (A.C.); 10 Division of Rheumatology, School of Medicine, Selcuk University, 42250 Konya, Turkey; sema.yilmaz@selcuk.edu.tr; 11 Division of Rheumatology, School of Medicine, Marmara University, 34854 Istanbul, Turkey; ginanc@marmara.edu.tr (N.I.); haner@marmara.edu.tr (H.D.); sule.yavuz@marmara.edu.tr (S.Y.); 12 Division of Rheumatology, School of Medicine, Inonu University, 44000 Malatya, Turkey; servet.yolbas@inonu.edu.tr; 13 Economics, Zitelab Aps, 2000 Copenhagen, Denmark; nielssteenkrogh@zitelab.dk; 14 Division of Rheumatology, School of Medicine, Bilim University, 34394 Istanbul, Turkey; neslihan.yilmaz@istanbulbilim.edu.tr; 15 Division of Rheumatology, School of Medicine, Yildirim Beyazit University, 06760 Ankara, Turkey; serten@aybu.edu.tr; 16 Division of Rheumatology, Istanbul Basaksehir Cam and Sakura Hospital, 34758 Istanbul, Turkey; cemalbes@hotmail.com

**Keywords:** Ankylosing spondylitis, biosimilar, CT-P13, infliximab, cohort, TURKBIO

## Abstract

**Background/Objectives:** CT-P13 is a biosimilar version of infliximab, a monoclonal antibody. In individuals with ankylosing spondylitis (AS), CT-P13 has been shown to be effective and to have a well-tolerated safety profile. The aim of this study was to evaluate the long-term drug persistence, safety, and efficacy of infliximab biosimilar CT-P13 in patients with AS undergoing first-line (1st-line) and later (≥2nd-line) treatment in clinical practice. **Methods:** We performed an observational cohort study that included AS patients based on the biological drug database in the TURKBIO Registry between 2014 and 2021. The patients were divided into two groups: those receiving CT-P13 as first-line treatment or as a switch (≥2nd-line) from another TNF inhibitor (TNFi). Standard disease activity metrics were used to assess the effectiveness of CT-P13, and drug retention rates were investigated. **Results:** There were 179 AS patients using CT-P13 (47.4% male, mean age: 42.9 ± 11.3 years). Of these patients, 123 (68.7%) were receiving CT-P13 as a first-line treatment. The mean length of treatment was 3.5 years. CT-P13 drug retention rates in the general patient population were 58.6% and 48.2% in the first-line and ≥second-line treatment, respectively, after 3 years of follow-up. The most common reason for CT-P13 treatment discontinuation was lack of efficacy. The first-line CT-P13 group had statistically substantially higher ASAS20/40 response rates at three and six months. Nonetheless, both groups’ response rates at one year were comparable. **Conclusions:** In this real-world data analysis, AS patients who were TNFi naïve (1st-line) and subsequently treated (≥2nd-line) with CT-P13 showed encouraging drug retention rates with acceptable long-term effectiveness and safety.

## 1. Introduction

Ankylosing spondylitis (AS), the most common and prototypical form of spondyloarthritis (SpA), is a chronic inflammatory disease characterized by axial and peripheral joint involvement [1]. The prevalence of AS is reported to be 0.12–1% in Europe and 0.55% in the United States of America. In an epidemiological study conducted in Turkey, the prevalence of AS was found to be 0.49% [2].

CT-P13 (Remsima^®^), a biosimilar of infliximab used to treat active rheumatic diseases, has been added to the list of biological therapies [3]. CT-P13 is a human murine chimeric immunoglobulin gamma 1 monoclonal antibody that is biosimilar to infliximab [4].

A Phase I, multicenter, double-blind, randomized controlled trial (RCT) found evidence of pharmacokinetic equivalence between CT-P13 and reference infliximab (RI) in 250 patients with AS (PLANETAS) [5]. The PLANETAS study found that improvements in AS signs and symptoms, as evaluated by the Assessment of SpA International Society response (ASAS 20/40) rates at week 30, did not differ significantly between CT-P13 and RI (70.5% and 51.8% versus 72.4% and 47.4%, respectively). It was noted that there was no significant difference in efficacy measures assessing disease activity between the RI and its CT-P13, including the AS Disease Activity Score (ASDAS)-CRP level, Bath Ankylosing Spondylitis Disease Activity Index (BASDAI), Bath Ankylosing Spondylitis Metrology Index (BASMI), and Bath Ankylosing Spondylitis Functional Index (BASFI) [6]. Results obtained from the PLANETAS extension study demonstrated that CT-P13’s efficacy and safety in patients treated for 54 and 102 weeks were comparable to patients who switched from RI to CT-P13, and response rates remained stable and did not differ significantly. In patients with active AS, the pharmacokinetic, efficacy, and safety profiles of CT-P13 and RI are equivalent, according to the study results [7].

Using information from AS patients registered in the Turkish Biological (TURKBIO) database, we sought to assess the efficacy, safety, and treatment adherence of first-line (1st-line) and second or subsequent (≥2nd-line) CT-P13 therapy.

## 2. Materials and Methods

### 2.1. Study Population

The data used in this investigation came from the TURKBIO database in Turkey, which was created in 2011 as the Turkish version of the Danish DANBIO Rheumatological database [2]. The main purpose of this large national registry is to collect and analyze information from patients receiving biologic and targeted synthetic disease-modifying antirheumatic drugs (bDMARDs and tsDMARDs) for rheumatic diseases. It includes adult (≥18 years) patients with AS. Exclusion criteria were as follows: (i) patients without follow-up data; (ii) patients who withdrew informed consent. A total of 179 patients who satisfied the ASAS criteria [1] for AS in the TURKBIO registry database between December 2014 and December 2021 were categorized as those receiving CT-P13 (5 mg/kg by intravenous infusion over 2 h at 0, 2, and 6 weeks, then every 6–8 weeks) therapy as a 1st-line or as a transition from another TNF inhibitor (TNFi) (≥2nd-line). All patients gave informed consent prior to any data collection, and the local ethics committee accepted the research methodology and data collecting form.

### 2.2. Data Collection and Outcomes

Data on clinical indicators, such as symptoms upon diagnosis, laboratory findings, disease activity markers, and other follow-up measures (BASDAI, BASFI, ASDAS-CRP, and Health Assessment Questionnaire–Spondylitis (HAQ)) [8,9,10,11], as well as information on past and present therapies and adverse effects, were evaluated and compared for the two groups. The effectiveness was assessed using the patient global assessment (GA), ASAS 20/40 response criteria [12], BASDAI, and ASDAS-CRP. The data about the treatment included details of the reasons for discontinuing treatment. Discontinuation of treatment was defined as the permanent discontinuation of biological agents. Exclusion of observations occurred when treatment was stopped for reasons other than adverse events (AE) and a lack of efficacy, such as remission or alternative intentions to get pregnant, poor adherence, or loss of follow-up. We evaluated and compared the reasons for cessation, which included AEs, primary failure, and secondary failure. Treatment failure was classified into two categories based on when it happened: primary failure (no effectiveness or response within six months of beginning therapy) and secondary failure (efficacy or response within six months but lost over time).

### 2.3. Ethics

The TURKBIO database project has been approved as a phase IV observational trial by the Local Ethics Committee and the Turkish Ministry of Health’s Drug Regulatory Agency (Decision No: 20.06.2013/253). Date: 20 June 2013). Written permission was obtained from the participants for the TURKBIO registry, and the principles specified in the Declaration of Helsinki were adhered to in the conduct of the research.

### 2.4. Statistical Analysis

For the analysis of the variables, IBM Corporation, Armonk, NY, USA, provided SPSS 25.0 software. The Shapiro–Wilk test was utilized to assess the conformity of univariate data to the normal distribution, the Mardia (Dornik and Hansen omnibus) test was used to assess the conformance to multivariate normal distribution, and the Levene test was employed to determine the homogeneity of variance. Appropriate parametric and nonparametric analyses were performed in accordance with the normal distribution and homogeneity of variance of the data. Quantitative variables were shown in the tables as the mean ± sd (standard deviation) and median (interquartile range [IQR25-75]), while categorical variables were shown as n (%). The drug retention was evaluated using the Kaplan–Meier curves. A log–rank test was performed to show the differences in the drug retention rates. The power analysis with a medium-sized effect (d = 0.5), significance level (α = 0.05), and 80% power (1 − β = 0.80) showed that a minimum of 55 participants were needed for each group. The total sample size for the two groups was approximately 110 participants. A *p*-value of less than 0.05 was considered significant in the analysis of the variables, which was conducted at a 95% confidence level.

## 3. Results

### 3.1. Patient Characteristics

A total of 179 patients receiving CT-P13 treatment were included in this study, 123 (68.7%) of whom were receiving it in first-line and 56 (31.3%) in ≥second-line treatment. The mean age for the first-line and ≥second-line treatment groups was 42.6 ± 11.1 and 43.7 ± 11.8 years, respectively (*p* = 0.094). Both gender and smoking status did not differ statistically significantly between the two groups. Compared with patients treated with CT-P13 in the first-line setting, those receiving CT-P13 in the ≥second-line had a longer disease duration (8.9 years vs. 13.7 years, respectively, *p* = 0.083) (Table 1). In all groups, there was a comparable distribution of extra-musculoskeletal abnormalities, such as psoriasis, uveitis, and inflammatory bowel disease (IBD), as well as peripheral arthritis, enthesitis, and dactylitis. Between the groups, there was no difference in the family history of SpA and associated disorders. The frequency of prior treatment use, including corticosteroids and NSAIDs, was higher in the ≥second-line therapy groups (*p* = 0.001 and *p* = 0.036, respectively) (Table 1).

### 3.2. Duration of Treatment

During the course of the more than three-year follow-up, no discernible differences were seen between the patient groups. The median IQR treatment duration was 46 (19–70) months for the first-line group and 35.5 (16–55) months for the ≥second-line group (*p* = 0.087). Overall, the most common reason for discontinuing CT-P13 treatment in the first-line and ≥second-line groups was a lack of efficacy (32.5% and 41.0%, respectively). In both groups, the rates of permanent discontinuation due to AEs were similar (1.6% and 1.7%, respectively). Adalimumab (28%) and etanercept (19.2%) were the most commonly used bDMARDs before CT-P13, followed by certolizumab, infliximab, and golimumab.

### 3.3. Drug Efficacy

During the first year of follow-up, there was no statistically significant difference in the disease activity ratings between the two groups (Table 2). At 3 and 6 months, the ASAS20/40 response rates were statistically considerably higher in the first-line CT-P13 group. Significant improvements in ASAS20/40 response rates were achieved in the majority of both patient groups in the first year of treatment. However, response rates at 12 months were similar between both groups (Table 3).

### 3.4. Drug Retention

**Figure 1:**
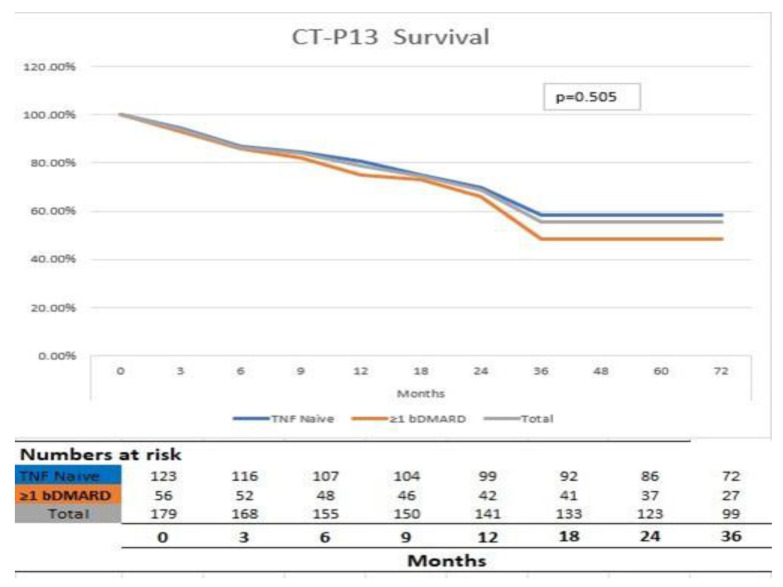
CT-P13 drug survival graphic in 1st-line and ≥2nd-line groups.

Drug retention rates, the main finding of our study, were shown to be comparable in the two groups. Before, in the general patient population, the three-year retention rate (95% CI) was 58.6% for the first-line group and 48.2% for the ≥second-line group (*p* > 0.05). The patient groups’ drug retention is summarized in Figure 1.

### 3.5. Safety

CT-P13 was well tolerated during long-term treatment. In both groups, the rates of discontinuation due to AEs were similar (Table 4). During the follow-up period, four (3.2%) of the first-line group and one (1.7%) of the ≥second-line group drugs were changed due to allergic side effects. During the first 3 years of CT-P13 treatment, one patient in the first-line group developed breast cancer, and one patient developed cervix cancer. In addition, treatment was discontinued in one patient due to drug-induced lupus. While there was no patient developing cancer in the ≥second-line group, CT-P13 treatment was terminated in one patient due to severe knee osteomyelitis.

### 3.6. Factors Associated with Retention Rates on CT-P13 in All AS Patients

The Cox regression analysis of drug discontinuation showed that the presence of enthesitis (HR: 0.19; 95% CI; 0.04–0.82, *p* = 0.026), a higher BMI (HR: 1.22; 95% CI 1.02–1.46, *p* = 0.026), and the baseline BASFI (HR: 0.91; 95% CI 0.86–0.96, *p* = 0.002) were associated with the risk of first-line CT-P13 treatment discontinuation in AS. Therefore, baseline ASDAS-CRP (HR: 0.08; 95% CI; 0.01–0.53, *p* = 0.008) and current methotrexate treatment (HR: 0.06; 95% CI 0.11–0.35, *p* = 0.002) were associated with the risk of second-line CT-P13 treatment discontinuation in AS (Table 5).

## 4. Discussion

This prospective, registry-based, observational study found encouraging drug retention rates in AS patients receiving CT-P13 as first-line or ≥second-line treatment for 3 years. CT-P13 had reasonable long-term efficacy and was tolerated with similar efficacy in the first-line and ≥second-line groups, according to our findings. Significant improvements in ASAS20/40 response rates were achieved in the majority of both patient groups in the first year of treatment. Lack of efficacy was the most frequent cause of CT-P13 therapy cessation. The rate of serious AEs for which treatment was permanently stopped was 2.2%.

The European Medical Association (EMA) approved CT-P13, marketed as Remsima, as the first biosimilar for patients with rheumatic diseases in 2013 [13]. Data from the original AS studies demonstrated that the transition from RI to biosimilar infliximab had similar effects on safety and efficacy [3,14,15]. CT-P13 was generally well tolerated in patients with AS for up to 54 weeks of treatment, with a tolerability profile similar to the RI [14]. Treatment-related severe AEs occurred in 3.1% of CT-P13 patients and 4.1% of patients receiving RI. Real-world studies have also found that switching from RI to CT-P13 is generally well tolerated in patients with rheumatic diseases with no safety signals.

Our CT-P13 treatment results are consistent with previous RI studies [16,17,18,19,20,21]. In registry-based studies conducted in Spain [16] and the Czech Republic [20], lack of efficacy was the most common reason for the discontinuation of RI. Of the 244 individuals with AS treated with CT-P13 in a Korean research study, 83.2% received the drug as their first line of therapy [22]. In the KOBIO registry, the drug retention rate tended to be longer in patients receiving CT-P13 as first-line treatment than in patients receiving CT-P13 in subsequent steps from 4 years of follow-up, but the differences between the groups were not statistically significant (log–rank *p* = 0.270). In our study, the drug retention rate at the 3-year follow-up was similar to the KOBIO registry study, and there was no statistically significant difference between the groups (log–rank *p* = 0.505). According to our research, inefficacy was the most frequent cause of cessation, and injection or infusion site responses were the most frequent AEs associated with CT-P13.

Real-world studies in rheumatology patient populations have revealed comparable efficacy and tolerability outcomes after switching from the RI to CT-P13, and this experience is supported by studies in IBD patients [23,24,25]. The body of research on the effectiveness and safety of CT-P13 therapy in individuals with AS seems to allay worries as more real-world data become available. Biosimilars provide comparable efficacy and safety to the original molecules, as well as significant economic benefits due to their lower cost when compared to the original molecule. Although there is currently no cost study specifically examining the use of CT-P13 in the management of AS patients, a number of studies assessing the financial effects of moving from RI to CT-P13 for a variety of purposes offer strong proof of CT-P13 cost reductions [26,27,28,29].

The ASAS20 response in 325 AS patients receiving certolizumab treatment was examined by Landewe et al. [30]. There was no discernible difference between patients with AS and those with non-radiographic axial SpA at week 12, according to the ASAS20 response rate, which was 58–64%. This is consistent with our findings that the ASAS20 response rate at 12 months was 76.5%. Real-life data have also found that switching from RI to CT-P13 is generally well tolerated in patients with rheumatic diseases, with no safety signals [31,32].

As previously mentioned [6,7], PLANETAS’s efficacy results at weeks 30 and 54 were similar to those of the infliximab arm of ASSERT (Ankylosing Spondylitis Study for the Evaluation of Recombinant infliximab Treatment) as well as another pivotal study that used a placebo and examined the infliximab reference product (RP) in AS over 24 and 54 weeks [33,34,35]. At week 102, the conclusion of the PLANETAS extension, the ASAS response rates in the maintenance arm were similar to those in week 102 of ASSERT (ASAS20: 80.7% vs. 73.9%; ASAS40: 63.9% vs. 59.4%) [36]. In our study, both groups’ ASAS response rates at one year were comparable (ASAS20: 76.5% vs. 70.6%; ASAS40: 61.8% vs. 41.2%).

These immunomodulators can act throughout the body and affect different organ systems. They can have both benefits and potential side effects. By weakening the immune response, they can make the body more susceptible to infection. For example, TNFi has been associated with increased levels of Treponema denticola, a periodontal pathogen. This suggests that it may have a significant effect on periodontal health [37]. Follow-up results of more than 3 years in the TURKBIO registry have provided important information regarding long-term treatment outcomes with CT-P13. We think that the availability of such data may assist in allaying physicians’ concerns about starting or transitioning patients to a biosimilar, including worries about possible immunogenicity changes, unanticipated AEs, or loss of effectiveness [38]. Like other nations, Turkey’s total AS treatment costs are heavily impacted by biological therapies. By resolving these issues, rheumatologic biologic therapy costs can be greatly decreased, and patient access to these treatments can be enhanced [26,28,39].

The TURKBIO registry prospectively collects real-world patient data without the typical protocol patient selection or mandatory treatment used in clinical trials and is, therefore, more representative of everyday clinical practice [40,41]. This means that our results should be consistent with the outcomes of AS patients treated with CT-P13 in a real-world clinical setting. In our study, the CT-P13 treatment of TNFi-naive patients provided important information about the evaluation of drug response.

This study’s primary strength is the real-world data it presents from Turkish individuals with AS receiving biological therapy. Other strengths of this study include the long follow-up period, the prospective nature of the TURKBIO registry, and the relatively large patient population studied. Nevertheless, the research did not look at the effectiveness of patients who switched from CT-P13 to infliximab RP (or vice versa). A constraint on the interpretation of effectiveness data is the lack of standardization among participating centers for some measures, such as CRP. Potential limitations include the fact that the data were collected from a registry, which may introduce the potential for selection bias and missing information. In addition, the study focuses primarily on the Turkish population, and the results may not be fully generalizable to other populations due to possible geographical and racial differences.

## 5. Conclusions

In recent years, biological therapies have played an important role in the treatment of inflammatory rheumatic diseases, particularly AS. Infliximab is a TNF-alpha inhibitor that is widely used in the treatment of these diseases. However, the use of biosimilars is becoming increasingly important in view of the cost of biological medicines. In this context, CT-P13 has been developed as a biosimilar of infliximab and has been shown to be pharmacokinetically and therapeutically equivalent to the reference product. In our study, CT-P13 treatment was evaluated in Turkish AS patients who were TNFi-naive (1st-line) and had a history of ≥1 TNFi (≥2nd-line). Observations in these patients provided reasonable long-term efficacy and safety data, as well as encouraging drug rates and durations. These findings suggest that CT-P13 is an effective and safe option for AS patients requiring RI therapy and that such biosimilars may offer a more economical alternative, particularly in terms of access to biologic therapy.

## Figures and Tables

**Table 1:** Demographic and clinical characteristics of AS patients.

	CT-P13		*p*-Value
	1st-Line	≥2nd-Line	
	(N = 123)	(N = 56)	
Age, years	42.6 ± 11.1	43.7 ± 11.8	0.094
Disease duration, years	8.9 ± 5.4	13.7 ± 9.2	0.083
HLA-B27 positive	48 (46.6)	23 (50)	0.702
Male	59 (47.9)	26 (46.4)	0.873
Smoking status, ever	65 (52.8)	23 (50)	0.529
BMI, kg/m^2^	27.7 ± 5.1	27.9 ± 6.5	0.826
Family history	30 (24.6)	20 (35.7)	0.397
Peripheral arthritis,	34 (27.9)	18 (32.1)	0.795
Uveitis	27 (22.1)	19 (33.9)	0.089
Enthesitis	39 (32.0)	31 (55.3)	0.092
Dactylitis	3 (2.4)	0 (0.0)	0.239
Psoriasis	7 (5.6)	7 (12.5)	0.574
Inflammatory bowel disease	6 (4.8)	8 (14.2)	0.604
Prior treatment			
Methotrexate	19 (15.6)	15 (26.7)	0.080
Sulfasalazine	72 (59)	40 (71.4)	0.169
Corticosteroid	15 (12.3)	19 (33.9)	0.001
NSAIDs	97 (79.5)	37 (66)	0.036
Concomitant treatment			
Methotrexate	9 (7.4)	9 (16)	0.081
Sulfasalazine	21 (17.2)	8 (14.2)	0.591
Corticosteroid	5 (4.2)	4 (7.1)	1.000
NSAIDs	50 (41)	26 (46.4)	0.559

Continuous variables were presented as mean SD, and categorical variables were presented as number (%). BMI, body mass index; HLA-B27, human leukocyte antigen-B27; NSAIDs, nonsteroidal anti-inflammatory drugs.

**Table 2:** Disease activity of patients using 1st-line and ≥2nd-line CT-P13 at 12-month follow-up.

	First-Line (n = 123)	≥Second-Line (n = 56)	
			*p*-Value
	Median (IQR 25–75)	Median (IQR 25–75)	
ASDAS-CRP (Baseline)	3.2 (2.8–3.9)	3.3 (2.9–3.9)	0.440
ASDAS-CRP (12th month)	1.6 (1.2–2)	1.7 (1.4–2.6)	0.067
BASDAI (Baseline)	58 (50–65)	55.5 (48–66)	0.663
BASDAI (12th month)	13.5 (8–24)	18 (6–42)	0.147
BASFI (Baseline)	41 (26–56)	47.5 (29–62)	0.166
BASFI (12th month)	14.5 (0.5–25.5)	17 (4–32)	0.120
CRP (Baseline)	8.8 (3–22)	7 (4–18.4)	0.898
CRP (12th month)	4.7 (2–7.4)	4.8 (3–10.4)	0.073
ESR (Baseline)	22 (10–33)	21 (7.5–34.5)	0.356
ESR (12th month)	11 (5–24)	15 (5–31)	0.165
VAS Pain (Baseline)	60 (50–75)	62.5 (51–78)	0.401
VAS Pain (12th month)	15 (0–30)	24.5 (0–48)	0.091
VAS Global (Baseline)	60 (51–70)	64.5 (53–80)	0.351
VAS Global (12th month)	20 (0–30.5)	22.5 (0–50)	0.114
Vas Physician (Baseline)	49 (28–61)	42 (32–60)	0.999
Vas Physician (12th month)	10 (0–30)	15 (0–34)	0.704
HAQ (Baseline)	1 (0.6–1.5)	1.1 (0.7–1.7)	0.131
HAQ (12th month)	0.2 (0–0.6)	0.5 (0–0.7)	0.503

ASDAS, Ankylosing Spondylitis Disease Activity Score; BASDAI, Bath Ankylosing Spondylitis Disease Activity Index; ESR, Erythrocyte Sedimentation Rate; VAS, Visual Analog Scale; HAQ, The Health Assessment Questionnaire; IQR, Interquartile range; n, number.

**Table 3:** ASAS20/40 response rates of patients.

CTP-13				
	No of Patients with Available Data, n	1st-Line	≥2nd-Line	*p* Value
ASAS 20, %				
3 months	150 (102/48)	76.5	50	0.006
6 months	115 (81/34)	82.7	44.1	0.008
12 months	85 (68/17)	76.5	70.6	0.262
ASAS 40, %				
3 months	150 (102/48)	57.8	27.1	0.049
6 months	115 (81/34)	69.1	20.6	0.003
12 months	85 (68/17)	61.8	41.2	0.079

Categorical variables were presented as number (%). ASAS, Assessment in Ankylosing Spondylitis response criteria.

**Table 4:** Safety profile of CT-P13.

		1st-Line (n = 123)	≥2nd-Line (n = 56)
		n (%)	n (%)
	Switch to another biologic		
Drug Retention	Primer inefficacy	7 (14)	6 (23)
	Secondary inefficacy	33 (66)	17 (65.3)
	SAE	3 (2.4)	1 (1.7)
	Mild-Moderate Allergic Reactions	4 (3.2)	1 (1.7)
	Permanently Stopped		
SAE	Cancer *	2 (1.6)	0 (0)
	Osteomyelitis	0 (0)	1 (1.7)
	Drug Associated Lupus	1 (0.8)	0 (0)

Categorical variables were presented as number (%). * Early-stage breast cancer (n = 1) and early-stage cervix cancer (n = 1); SAE, Severe Adverse Event.

**Table 5:** Factors associated with CT-P13 discontinuation in 1st-line and ≥2nd-line groups.

	1st-Line				≥2nd-Line			
	Univariable Analysis		Multivariable Analysis ^a^		Univariable Analysis		Multivariable Analysis ^a^	
	HR (95% CI)	*p* Value	HR (95% CI)	*p* Value	HR (95% CI)	*p* Value	HR (95% CI)	*p* Value
Age, years	0.99 (0.95–1.02)	0.624			0.97 (0.93–1.00)	0.110		
Male, sex	1.17 (0.52–2.64)	0.701			1.22 (0.56–2.64)	0.610		
Disease duration	1.04 (0.97–1.12)	0.212			0.97 (0.93–1.02)	0.280		
Current smoker	0.67 (0.26–1.25)	0.423			1.63 (0.66–3.98)	0.281		
BMI, kg/m^2^	1.03 (0.94–1.13)	0.449	1.22 (1.02–1.46)	0.026	0.96 (0.88–1.03)	0.302		
Peripheral arthritis	0.54 (0.24–1.22)	0.141			0.73 (0.32–1.66)	0.463		
Enthesitis	2.18 (0.97–4.85)	0.057	0.19 (0.04–0.82)	0.026	1.00 (0.46–2.18)	0.990		
BASDAI (baseline)	0.98 (0.95–1.00)	0.180			0.99 (0.98–1.01)	0.780		
BASFI (baseline)	0.98 (0.96–1.00)	0.079	0.91 (0.86–0.96)	0.002	0.99 (0.98–1.01)	0.959		
ASDAS-CRP (baseline)	0.73 (0.45–1.19)	0.213			0.65 (0.39–1.08)	0.100	0.08 (0.01–0.53)	0.008
CRP (baseline)	1.00 (0.99–1.02)	0.358			0.98 (0.96–1.00)	0.204		
ESR (baseline)	0.99 (0.96–1.01)	0.390			0.98 (0.96–1.00)	0.194		
Current sulfasalazine	1.64 (0.49–5.52)	0.420			1.86 (0.44–7.94)	0.397		
Current methotrexate	0.98 (0.23–4.19)	0.985			0.37 (0.14–0.96)	0.042	0.06 (0.11–0.35)	0.002
Current NSAIDs	0.71 (0.32–1.61)	0.425			1.46 (0.65–3.26)	0.349		

^a^ Covariates with a *p* value < 0.1 in the univariable analysis included for multivariable analysis.

## Data Availability

The data that support the findings of this study are available from the Correspondence author.
